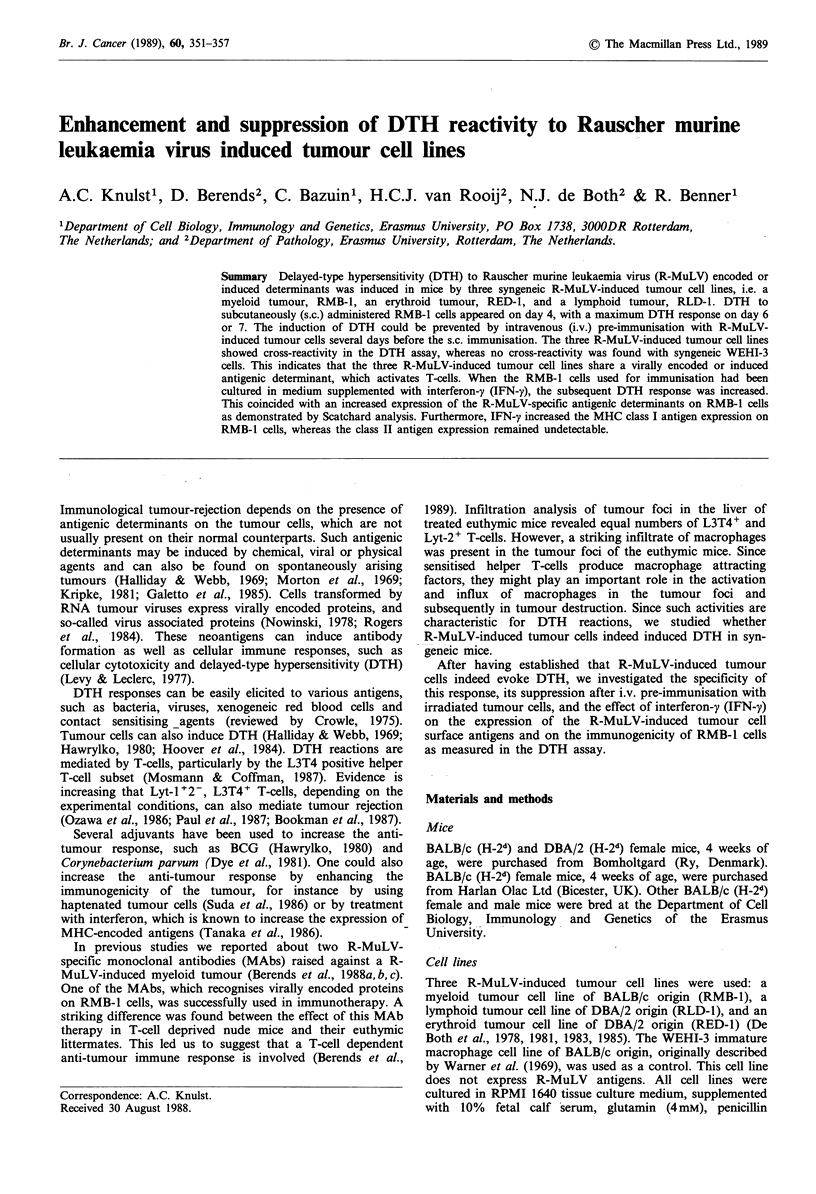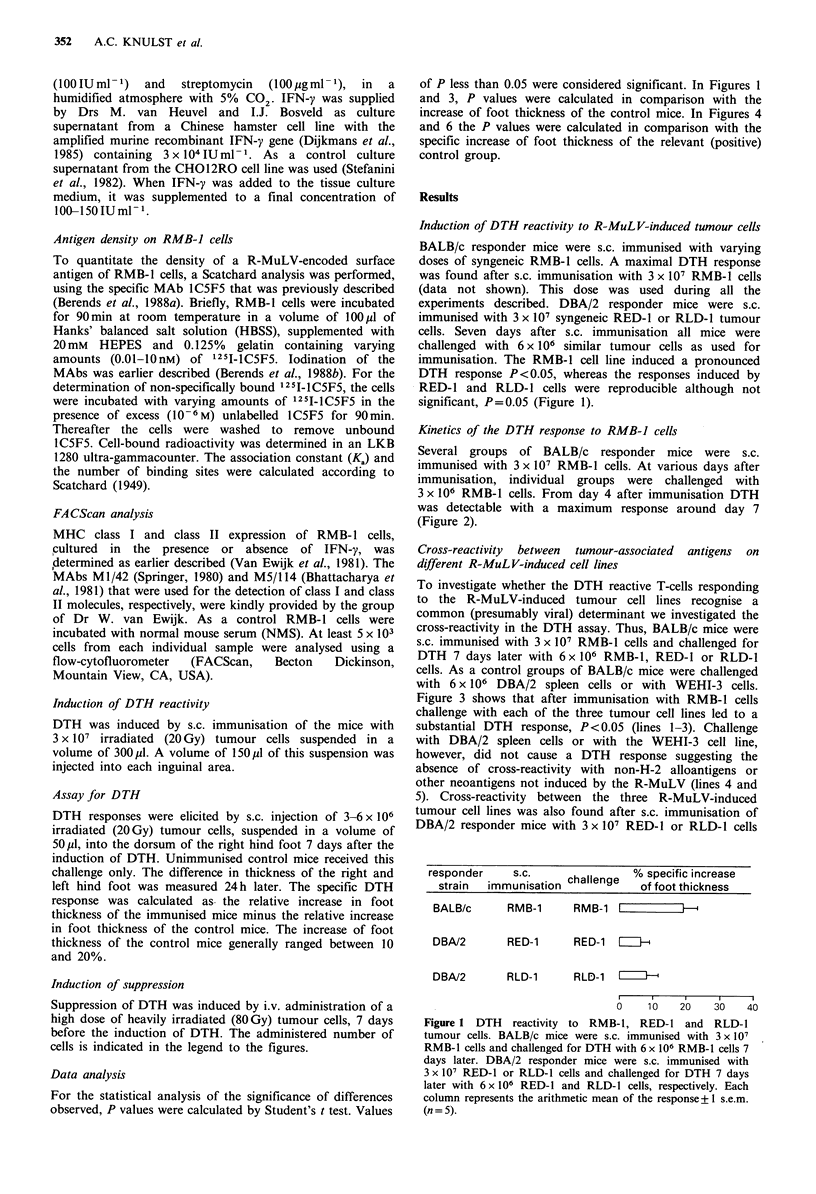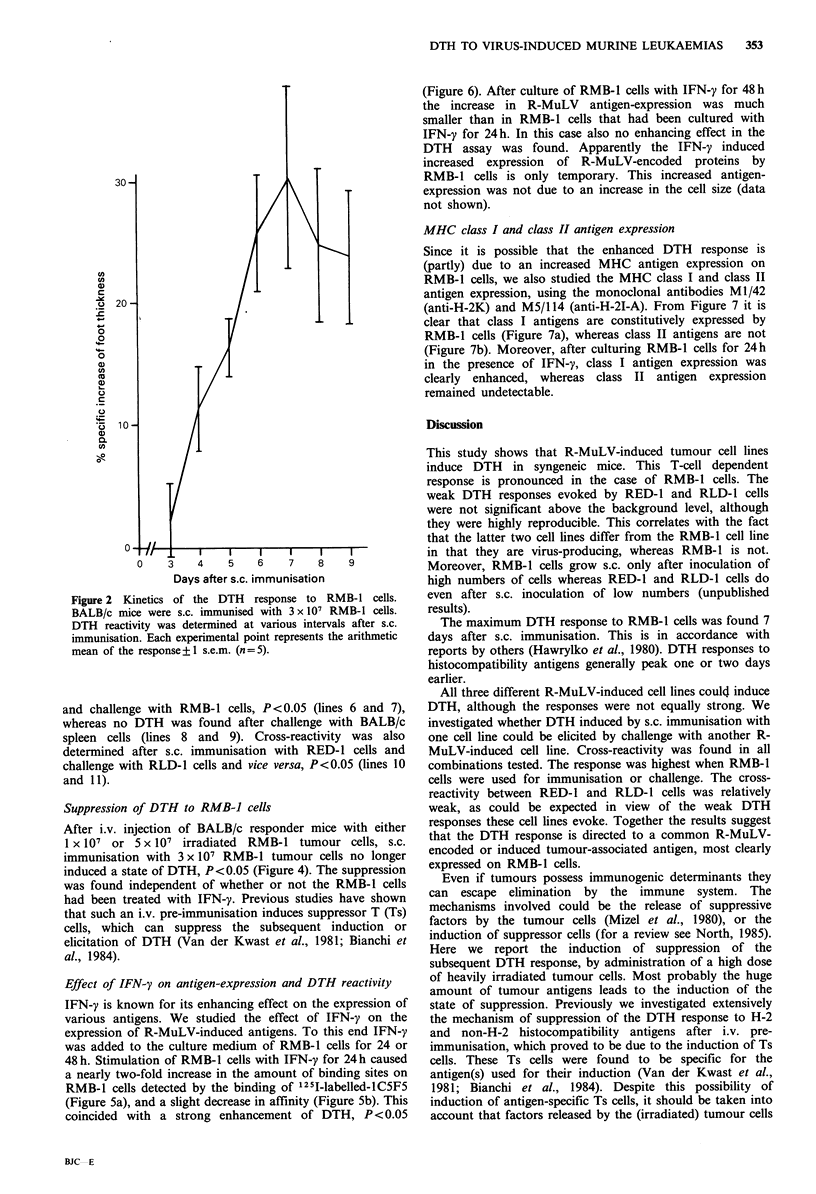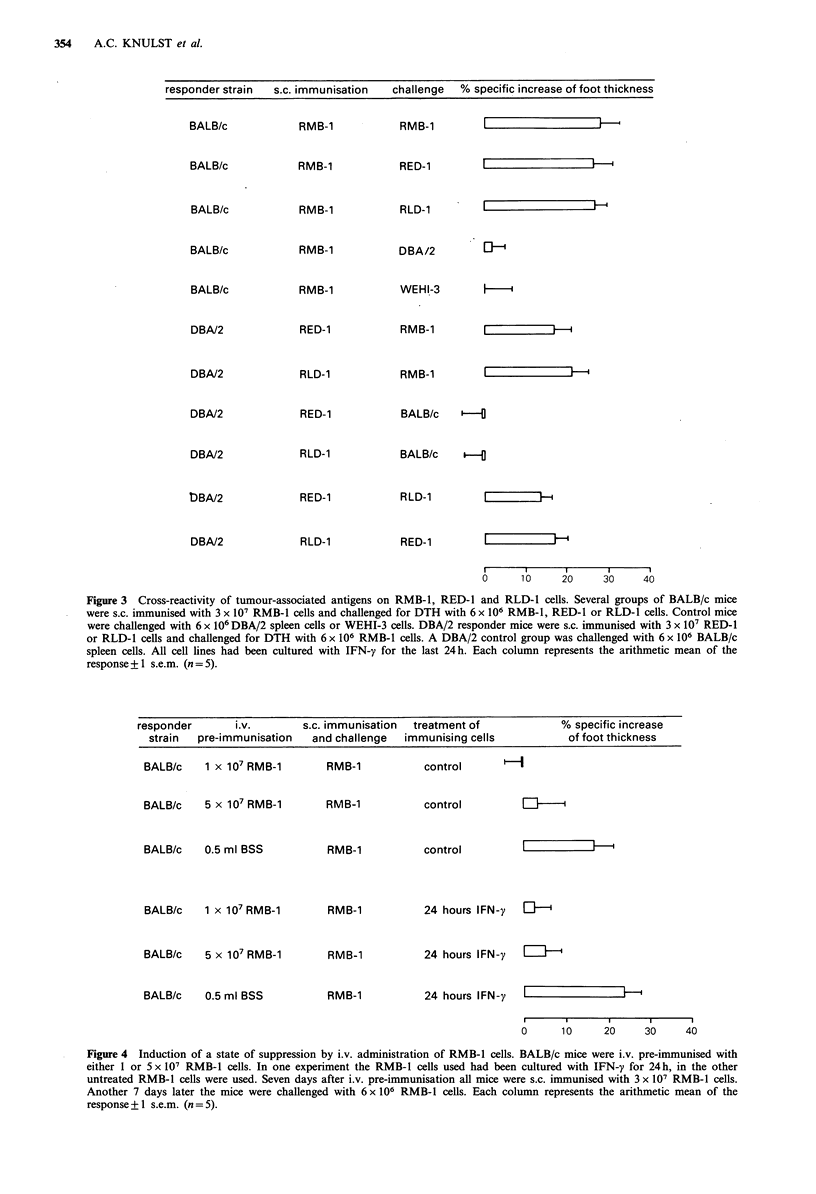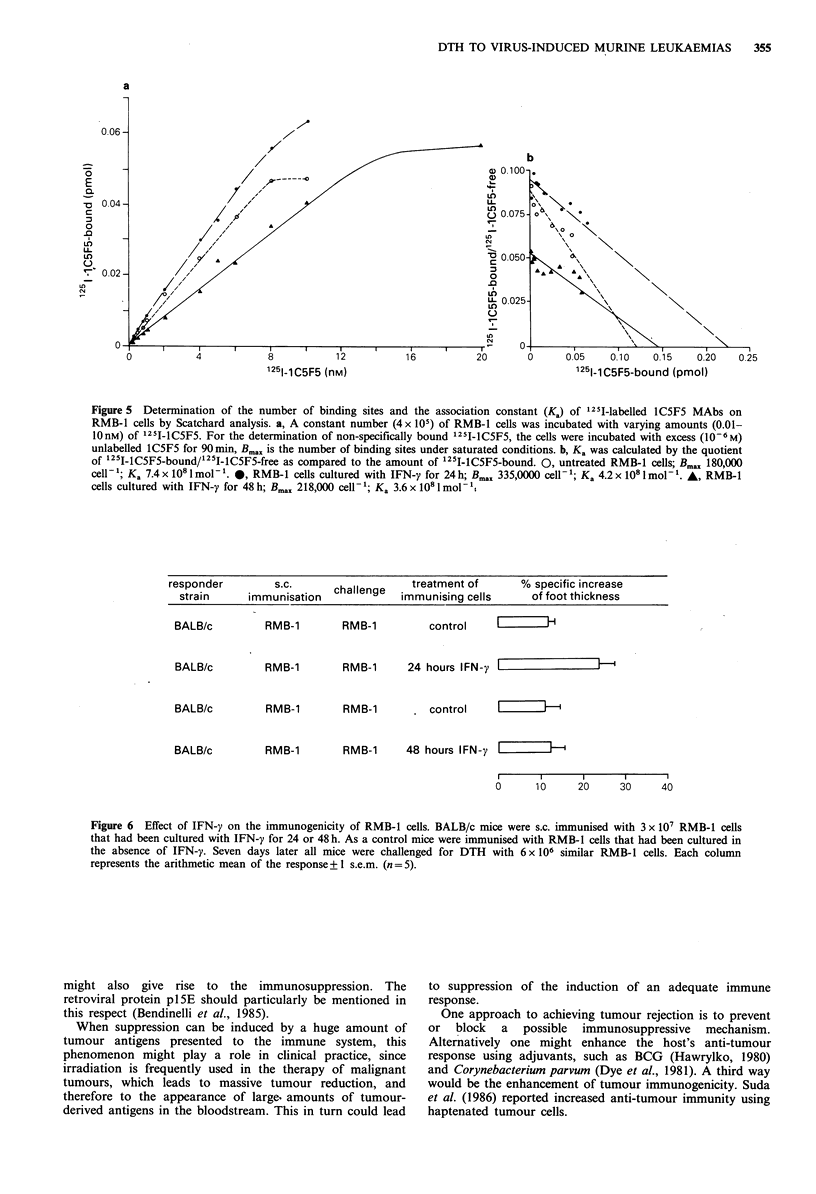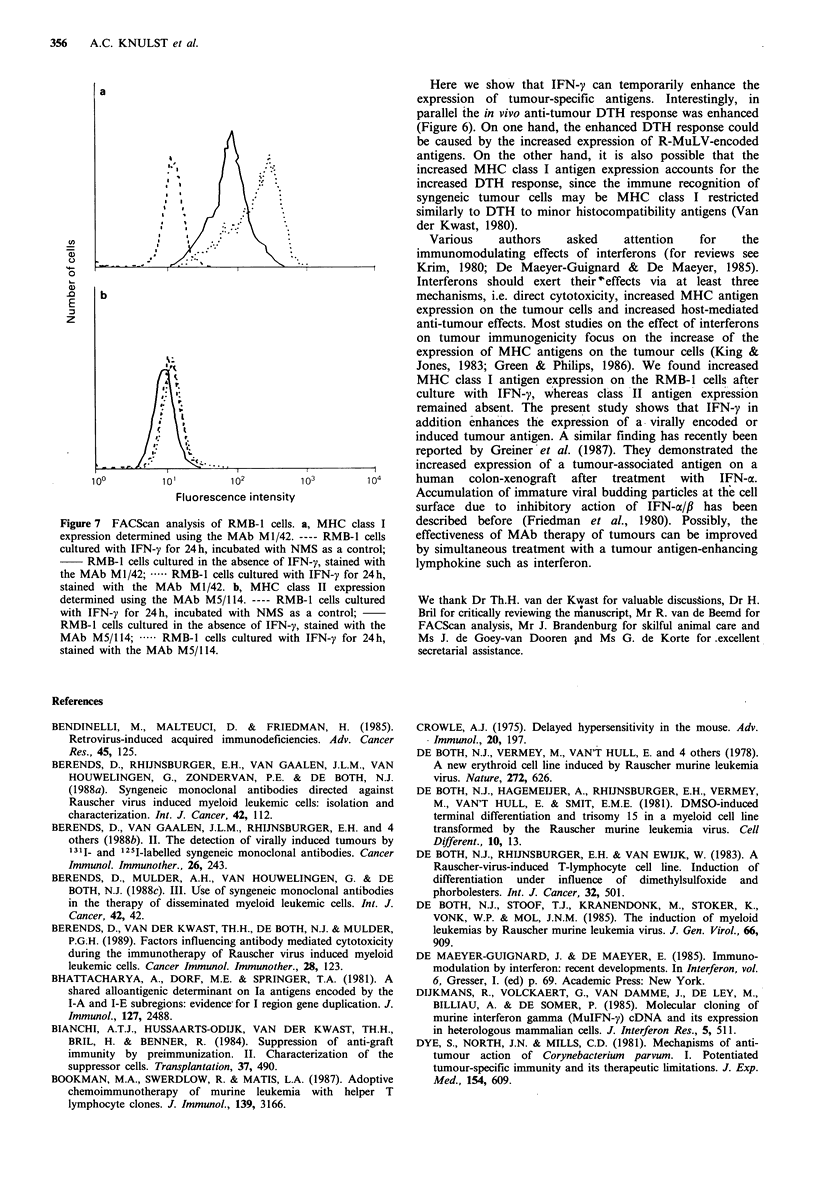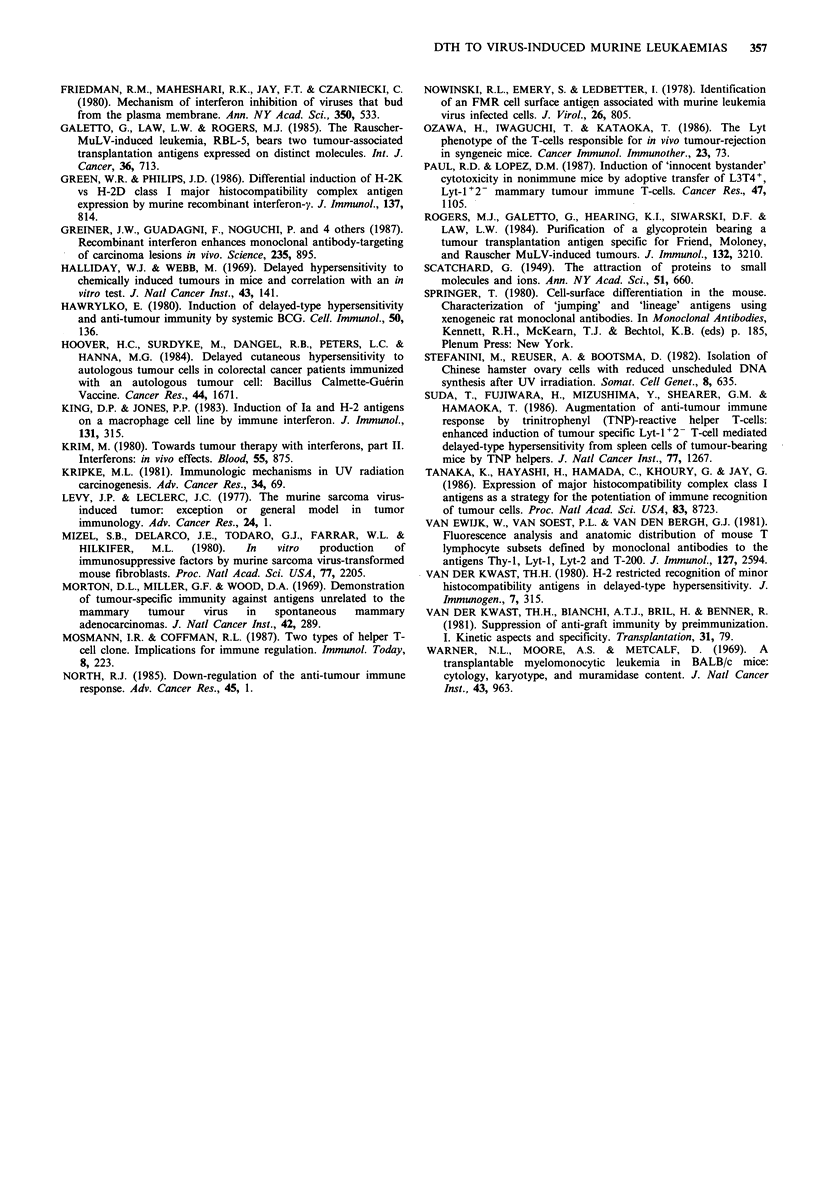# Enhancement and suppression of DTH reactivity to Rauscher murine leukaemia virus induced tumour cell lines.

**DOI:** 10.1038/bjc.1989.284

**Published:** 1989-09

**Authors:** A. C. Knulst, D. Berends, C. Bazuin, H. C. van Rooij, N. J. de Both, R. Benner

**Affiliations:** Department of Cell Biology, Erasmus University, Rotterdam, The Netherlands.

## Abstract

Delayed-type hypersensitivity (DTH) to Rauscher murine leukaemia virus (R-MuLV) encoded or induced determinants was induced in mice by three syngeneic R-MuLV-induced tumour cell lines, i.e. a myeloid tumour, RMB-1, an erythroid tumour, RED-1, and a lymphoid tumour, RLD-1. DTH to subcutaneously (s.c.) administered RMB-1 cells appeared on day 4, with a maximum DTH response on day 6 or 7. The induction of DTH could be prevented by intravenous (i.v.) pre-immunisation with R-MuLV-induced tumour cells several days before the s.c. immunisation. The three R-MuLV-induced tumour cell lines showed cross-reactivity in the DTH assay, whereas no cross-reactivity was found with syngeneic WEHI-3 cells. This indicates that the three R-MuLV-induced tumour cell lines share a virally encoded or induced antigenic determinant, which activates T-cells. When the RMB-1 cells used for immunisation had been cultured in medium supplemented with interferon-gamma (IFN-gamma), the subsequent DTH response was increased. This coincided with an increased expression of the R-MuLV-specific antigenic determinants on RMB-1 cells as demonstrated by Scatchard analysis. Furthermore, IFN-gamma increased the MHC class I antigen expression on RMB-1 cells, whereas the class II antigen expression remained undetectable.


Br. J. Cancer (1989), 60, 351–357 © The Macmillan Press Ltd., 1989

### Enhancement and suppression of DTH reactivity to Rauscher murine leukaemia virus induced tumour cell lines

A.C. Knulst[1], D. Berends[2], C. Bazuin[1], H.C.J. van Rooij[2], N.J. de Both[2] & R. Benner[1]

[1]*Department of Cell Biology, Immunology and Genetics, Erasmus University, PO Box 1738, 3000DR Rotterdam, The Netherlands; and* [2]*Department of Pathology, Erasmus University, Rotterdam, The Netherlands.*

**Summary** Delayed-type hypersensitivity (DTH) to Rauscher murine leukaemia virus (R-MuLV) encoded or induced determinants was induced in mice by three syngeneic R-MuLV-induced tumour cell lines, i.e. a myeloid tumour, RMB-1, an erythroid tumour, RED-1, and a lymphoid tumour, RLD-1. DTH to subcutaneously (s.c.) administered RMB-1 cells appeared on day 4, with a maximum DTH response on day 6 or 7. The induction of DTH could be prevented by intravenous (i.v.) pre-immunisation with R-MuLV-induced tumour cells several days before the s.c. immunisation. The three R-MuLV-induced tumour cell lines showed cross-reactivity in the DTH assay, whereas no cross-reactivity was found with syngeneic WEHI-3 cells. This indicates that the three R-MuLV-induced tumour cell lines share a virally encoded or induced antigenic determinant, which activates T-cells. When the RMB-1 cells used for immunisation had been cultured in medium supplemented with interferon-$\gamma$ (IFN-$\gamma$), the subsequent DTH response was increased. This coincided with an increased expression of the R-MuLV-specific antigenic determinants on RMB-1 cells as demonstrated by Scatchard analysis. Furthermore, IFN-$\gamma$ increased the MHC class I antigen expression on RMB-1 cells, whereas the class II antigen expression remained undetectable.

Immunological tumour-rejection depends on the presence of antigenic determinants on the tumour cells, which are not usually present on their normal counterparts. Such antigenic determinants may be induced by chemical, viral or physical agents and can also be found on spontaneously arising tumours (Halliday & Webb, 1969; Morton *et al.*, 1969; Kripke, 1981; Galetto *et al.*, 1985). Cells transformed by RNA tumour viruses express virally encoded proteins, and so-called virus associated proteins (Nowinski, 1978; Rogers *et al.*, 1984). These neoantigens can induce antibody formation as well as cellular immune responses, such as cellular cytotoxicity and delayed-type hypersensitivity (DTH) (Levy & Leclerc, 1977).

DTH responses can be easily elicited to various antigens, such as bacteria, viruses, xenogeneic red blood cells and contact sensitising agents (reviewed by Crowle, 1975). Tumour cells can also induce DTH (Halliday & Webb, 1969; Hawrylko, 1980; Hoover *et al.*, 1984). DTH reactions are mediated by T-cells, particularly by the L3T4 positive helper T-cell subset (Mosmann & Coffman, 1987). Evidence is increasing that Lyt-$1^+2^-$, L3T4$^+$ T-cells, depending on the experimental conditions, can also mediate tumour rejection (Ozawa *et al.*, 1986; Paul *et al.*, 1987; Bookman *et al.*, 1987).

Several adjuvants have been used to increase the anti-tumour response, such as BCG (Hawrylko, 1980) and *Corynebacterium parvum* (Dye *et al.*, 1981). One could also increase the anti-tumour response by enhancing the immunogenicity of the tumour, for instance by using haptenated tumour cells (Suda *et al.*, 1986) or by treatment with interferon, which is known to increase the expression of MHC-encoded antigens (Tanaka *et al.*, 1986).

In previous studies we reported about two R-MuLV-specific monoclonal antibodies (MAbs) raised against a R-MuLV-induced myeloid tumour (Berends *et al.*, 1988*a,b,c*). One of the MAbs, which recognises virally encoded proteins on RMB-1 cells, was successfully used in immunotherapy. A striking difference was found between the effect of this MAb therapy in T-cell deprived nude mice and their euthymic littermates. This led us to suggest that a T-cell dependent anti-tumour immune response is involved (Berends *et al.*, 1989). Infiltration analysis of tumour foci in the liver of treated euthymic mice revealed equal numbers of L3T4$^+$ and Lyt-2$^+$ T-cells. However, a striking infiltrate of macrophages was present in the tumour foci of the euthymic mice. Since sensitised helper T-cells produce macrophage attracting factors, they might play an important role in the activation and influx of macrophages in the tumour foci and subsequently in tumour destruction. Since such activities are characteristic for DTH reactions, we studied whether R-MuLV-induced tumour cells indeed induced DTH in syngeneic mice.

After having established that R-MuLV-induced tumour cells indeed evoke DTH, we investigated the specificity of this response, its suppression after i.v. pre-immunisation with irradiated tumour cells, and the effect of interferon-$\gamma$ (IFN-$\gamma$) on the expression of the R-MuLV-induced tumour cell surface antigens and on the immunogenicity of RMB-1 cells as measured in the DTH assay.

### Materials and methods

### *Mice*

BALB/c (H-$2^d$) and DBA/2 (H-$2^d$) female mice, 4 weeks of age, were purchased from Bomholtgard (Ry, Denmark). BALB/c (H-$2^d$) female mice, 4 weeks of age, were purchased from Harlan Olac Ltd (Bicester, UK). Other BALB/c (H-$2^d$) female and male mice were bred at the Department of Cell Biology, Immunology and Genetics of the Erasmus University.

### *Cell lines*

Three R-MuLV-induced tumour cell lines were used: a myeloid tumour cell line of BALB/c origin (RMB-1), a lymphoid tumour cell line of DBA/2 origin (RLD-1), and an erythroid tumour cell line of DBA/2 origin (RED-1) (De Both *et al.*, 1978, 1981, 1983, 1985). The WEHI-3 immature macrophage cell line of BALB/c origin, originally described by Warner *et al.* (1969), was used as a control. This cell line does not express R-MuLV antigens. All cell lines were cultured in RPMI 1640 tissue culture medium, supplemented with 10% fetal calf serum, glutamin (4 mM), penicillin

Correspondence: A.C. Knulst.
Received 30 August 1988.

(100 IU ml$^{-1}$) and streptomycin (100 $\mu$g ml$^{-1}$), in a humidified atmosphere with 5% $CO_2$. IFN-$\gamma$ was supplied by Drs M. van Heuvel and I.J. Bosveld as culture supernatant from a Chinese hamster cell line with the amplified murine recombinant IFN-$\gamma$ gene (Dijkmans *et al.*, 1985) containing $3 \times 10^4$ IU ml$^{-1}$. As a control culture supernatant from the CHO12RO cell line was used (Stefanini *et al.*, 1982). When IFN-$\gamma$ was added to the tissue culture medium, it was supplemented to a final concentration of 100–150 IU ml$^{-1}$.

*Antigen density on RMB-1 cells*

To quantitate the density of a R-MuLV-encoded surface antigen of RMB-1 cells, a Scatchard analysis was performed, using the specific MAb 1C5F5 that was previously described (Berends *et al.*, 1988*a*). Briefly, RMB-1 cells were incubated for 90 min at room temperature in a volume of 100 $\mu$l of Hanks' balanced salt solution (HBSS), supplemented with 20 mM HEPES and 0.125% gelatin containing varying amounts (0.01–10 nM) of $^{125}$I-1C5F5. Iodination of the MAbs was earlier described (Berends *et al.*, 1988*b*). For the determination of non-specifically bound $^{125}$I-1C5F5, the cells were incubated with varying amounts of $^{125}$I-1C5F5 in the presence of excess ($10^{-6}$ M) unlabelled 1C5F5 for 90 min. Thereafter the cells were washed to remove unbound 1C5F5. Cell-bound radioactivity was determined in an LKB 1280 ultra-gammacounter. The association constant ($K_a$) and the number of binding sites were calculated according to Scatchard (1949).

*FACScan analysis*

MHC class I and class II expression of RMB-1 cells, cultured in the presence or absence of IFN-$\gamma$, was determined as earlier described (Van Ewijk *et al.*, 1981). The MAbs M1/42 (Springer, 1980) and M5/114 (Bhattacharya *et al.*, 1981) that were used for the detection of class I and class II molecules, respectively, were kindly provided by the group of Dr W. van Ewijk. As a control RMB-1 cells were incubated with normal mouse serum (NMS). At least $5 \times 10^3$ cells from each individual sample were analysed using a flow-cytofluorometer (FACScan, Becton Dickinson, Mountain View, CA, USA).

*Induction of DTH reactivity*

DTH was induced by s.c. immunisation of the mice with $3 \times 10^7$ irradiated (20 Gy) tumour cells suspended in a volume of 300 $\mu$l. A volume of 150 $\mu$l of this suspension was injected into each inguinal area.

*Assay for DTH*

DTH responses were elicited by s.c. injection of $3–6 \times 10^6$ irradiated (20 Gy) tumour cells, suspended in a volume of 50 $\mu$l, into the dorsum of the right hind foot 7 days after the induction of DTH. Unimmunised control mice received this challenge only. The difference in thickness of the right and left hind foot was measured 24 h later. The specific DTH response was calculated as the relative increase in foot thickness of the immunised mice minus the relative increase in foot thickness of the control mice. The increase of foot thickness of the control mice generally ranged between 10 and 20%.

*Induction of suppression*

Suppression of DTH was induced by i.v. administration of a high dose of heavily irradiated (80 Gy) tumour cells, 7 days before the induction of DTH. The administered number of cells is indicated in the legend to the figures.

*Data analysis*

For the statistical analysis of the significance of differences observed, *P* values were calculated by Student's *t* test. Values of *P* less than 0.05 were considered significant. In Figures 1 and 3, *P* values were calculated in comparison with the increase of foot thickness of the control mice. In Figures 4 and 6 the *P* values were calculated in comparison with the specific increase of foot thickness of the relevant (positive) control group.

### Results

*Induction of DTH reactivity to R-MuLV-induced tumour cells*

BALB/c responder mice were s.c. immunised with varying doses of syngeneic RMB-1 cells. A maximal DTH response was found after s.c. immunisation with $3 \times 10^7$ RMB-1 cells (data not shown). This dose was used during all the experiments described. DBA/2 responder mice were s.c. immunised with $3 \times 10^7$ syngeneic RED-1 or RLD-1 tumour cells. Seven days after s.c. immunisation all mice were challenged with $6 \times 10^6$ similar tumour cells as used for immunisation. The RMB-1 cell line induced a pronounced DTH response $P<0.05$, whereas the responses induced by RED-1 and RLD-1 cells were reproducible although not significant, $P=0.05$ (Figure 1).

*Kinetics of the DTH response to RMB-1 cells*

Several groups of BALB/c responder mice were s.c. immunised with $3 \times 10^7$ RMB-1 cells. At various days after immunisation, individual groups were challenged with $3 \times 10^6$ RMB-1 cells. From day 4 after immunisation DTH was detectable with a maximum response around day 7 (Figure 2).

*Cross-reactivity between tumour-associated antigens on different R-MuLV-induced cell lines*

To investigate whether the DTH reactive T-cells responding to the R-MuLV-induced tumour cell lines recognise a common (presumably viral) determinant we investigated the cross-reactivity in the DTH assay. Thus, BALB/c mice were s.c. immunised with $3 \times 10^7$ RMB-1 cells and challenged for DTH 7 days later with $6 \times 10^6$ RMB-1, RED-1 or RLD-1 cells. As a control groups of BALB/c mice were challenged with $6 \times 10^6$ DBA/2 spleen cells or with WEHI-3 cells. Figure 3 shows that after immunisation with RMB-1 cells challenge with each of the three tumour cell lines led to a substantial DTH response, $P<0.05$ (lines 1–3). Challenge with DBA/2 spleen cells or with the WEHI-3 cell line, however, did not cause a DTH response suggesting the absence of cross-reactivity with non-H-2 alloantigens or other neoantigens not induced by the R-MuLV (lines 4 and 5). Cross-reactivity between the three R-MuLV-induced tumour cell lines was also found after s.c. immunisation of DBA/2 responder mice with $3 \times 10^7$ RED-1 or RLD-1 cells

**Figure 1** DTH reactivity to RMB-1, RED-1 and RLD-1 tumour cells. BALB/c mice were s.c. immunised with $3 \times 10^7$ RMB-1 cells and challenged for DTH with $6 \times 10^6$ RMB-1 cells 7 days later. DBA/2 responder mice were s.c. immunised with $3 \times 10^7$ RED-1 or RLD-1 cells and challenged for DTH 7 days later with $6 \times 10^6$ RED-1 and RLD-1 cells, respectively. Each column represents the arithmetic mean of the response $\pm$ 1 s.e.m. ($n=5$).

**Figure 2** Kinetics of the DTH response to RMB-1 cells. BALB/c mice were s.c. immunised with $3 \times 10^7$ RMB-1 cells. DTH reactivity was determined at various intervals after s.c. immunisation. Each experimental point represents the arithmetic mean of the response $\pm 1$ s.e.m. ($n = 5$).

and challenge with RMB-1 cells, $P < 0.05$ (lines 6 and 7), whereas no DTH was found after challenge with BALB/c spleen cells (lines 8 and 9). Cross-reactivity was also determined after s.c. immunisation with RED-1 cells and challenge with RLD-1 cells and *vice versa*, $P < 0.05$ (lines 10 and 11).

*Suppression of DTH to RMB-1 cells*

After i.v. injection of BALB/c responder mice with either $1 \times 10^7$ or $5 \times 10^7$ irradiated RMB-1 tumour cells, s.c. immunisation with $3 \times 10^7$ RMB-1 tumour cells no longer induced a state of DTH, $P < 0.05$ (Figure 4). The suppression was found independent of whether or not the RMB-1 cells had been treated with IFN-$\gamma$. Previous studies have shown that such an i.v. pre-immunisation induces suppressor T (Ts) cells, which can suppress the subsequent induction or elicitation of DTH (Van der Kwast *et al.*, 1981; Bianchi *et al.*, 1984).

*Effect of IFN-$\gamma$ on antigen-expression and DTH reactivity*

IFN-$\gamma$ is known for its enhancing effect on the expression of various antigens. We studied the effect of IFN-$\gamma$ on the expression of R-MuLV-induced antigens. To this end IFN-$\gamma$ was added to the culture medium of RMB-1 cells for 24 or 48 h. Stimulation of RMB-1 cells with IFN-$\gamma$ for 24 h caused a nearly two-fold increase in the amount of binding sites on RMB-1 cells detected by the binding of $^{125}$I-labelled-1C5F5 (Figure 5a), and a slight decrease in affinity (Figure 5b). This coincided with a strong enhancement of DTH, $P < 0.05$ (Figure 6). After culture of RMB-1 cells with IFN-$\gamma$ for 48 h the increase in R-MuLV antigen-expression was much smaller than in RMB-1 cells that had been cultured with IFN-$\gamma$ for 24 h. In this case also no enhancing effect in the DTH assay was found. Apparently the IFN-$\gamma$ induced increased expression of R-MuLV-encoded proteins by RMB-1 cells is only temporary. This increased antigen-expression was not due to an increase in the cell size (data not shown).

*MHC class I and class II antigen expression*

Since it is possible that the enhanced DTH response is (partly) due to an increased MHC antigen expression on RMB-1 cells, we also studied the MHC class I and class II antigen expression, using the monoclonal antibodies M1/42 (anti-H-2K) and M5/114 (anti-H-2I-A). From Figure 7 it is clear that class I antigens are constitutively expressed by RMB-1 cells (Figure 7a), whereas class II antigens are not (Figure 7b). Moreover, after culturing RMB-1 cells for 24 h in the presence of IFN-$\gamma$, class I antigen expression was clearly enhanced, whereas class II antigen expression remained undetectable.

### Discussion

This study shows that R-MuLV-induced tumour cell lines induce DTH in syngeneic mice. This T-cell dependent response is pronounced in the case of RMB-1 cells. The weak DTH responses evoked by RED-1 and RLD-1 cells were not significant above the background level, although they were highly reproducible. This correlates with the fact that the latter two cell lines differ from the RMB-1 cell line in that they are virus-producing, whereas RMB-1 is not. Moreover, RMB-1 cells grow s.c. only after inoculation of high numbers of cells whereas RED-1 and RLD-1 cells do even after s.c. inoculation of low numbers (unpublished results).

The maximum DTH response to RMB-1 cells was found 7 days after s.c. immunisation. This is in accordance with reports by others (Hawrylko *et al.*, 1980). DTH responses to histocompatibility antigens generally peak one or two days earlier.

All three different R-MuLV-induced cell lines could induce DTH, although the responses were not equally strong. We investigated whether DTH induced by s.c. immunisation with one cell line could be elicited by challenge with another R-MuLV-induced cell line. Cross-reactivity was found in all combinations tested. The response was highest when RMB-1 cells were used for immunisation or challenge. The cross-reactivity between RED-1 and RLD-1 cells was relatively weak, as could be expected in view of the weak DTH responses these cell lines evoke. Together the results suggest that the DTH response is directed to a common R-MuLV-encoded or induced tumour-associated antigen, most clearly expressed on RMB-1 cells.

Even if tumours possess immunogenic determinants they can escape elimination by the immune system. The mechanisms involved could be the release of suppressive factors by the tumour cells (Mizel *et al.*, 1980), or the induction of suppressor cells (for a review see North, 1985). Here we report the induction of suppression of the subsequent DTH response, by administration of a high dose of heavily irradiated tumour cells. Most probably the huge amount of tumour antigens leads to the induction of the state of suppression. Previously we investigated extensively the mechanism of suppression of the DTH response to H-2 and non-H-2 histocompatibility antigens after i.v. pre-immunisation, which proved to be due to the induction of Ts cells. These Ts cells were found to be specific for the antigen(s) used for their induction (Van der Kwast *et al.*, 1981; Bianchi *et al.*, 1984). Despite this possibility of induction of antigen-specific Ts cells, it should be taken into account that factors released by the (irradiated) tumour cells

**Figure 3** Cross-reactivity of tumour-associated antigens on RMB-1, RED-1 and RLD-1 cells. Several groups of BALB/c mice were s.c. immunised with $3 \times 10^7$ RMB-1 cells and challenged for DTH with $6 \times 10^6$ RMB-1, RED-1 or RLD-1 cells. Control mice were challenged with $6 \times 10^6$ DBA/2 spleen cells or WEHI-3 cells. DBA/2 responder mice were s.c. immunised with $3 \times 10^7$ RED-1 or RLD-1 cells and challenged for DTH with $6 \times 10^6$ RMB-1 cells. A DBA/2 control group was challenged with $6 \times 10^6$ BALB/c spleen cells. All cell lines had been cultured with IFN-$\gamma$ for the last 24 h. Each column represents the arithmetic mean of the response $\pm$ 1 s.e.m. ($n = 5$).

**Figure 4** Induction of a state of suppression by i.v. administration of RMB-1 cells. BALB/c mice were i.v. pre-immunised with either 1 or $5 \times 10^7$ RMB-1 cells. In one experiment the RMB-1 cells used had been cultured with IFN-$\gamma$ for 24 h, in the other untreated RMB-1 cells were used. Seven days after i.v. pre-immunisation all mice were s.c. immunised with $3 \times 10^7$ RMB-1 cells. Another 7 days later the mice were challenged with $6 \times 10^6$ RMB-1 cells. Each column represents the arithmetic mean of the response $\pm$ 1 s.e.m. ($n = 5$).

**Figure 5** Determination of the number of binding sites and the association constant ($K_a$) of $^{125}$I-labelled 1C5F5 MAbs on RMB-1 cells by Scatchard analysis. **a**, A constant number ($4\times10^5$) of RMB-1 cells was incubated with varying amounts (0.01–10 nM) of $^{125}$I-1C5F5. For the determination of non-specifically bound $^{125}$I-1C5F5, the cells were incubated with excess ($10^{-6}$ M) unlabelled 1C5F5 for 90 min, $B_{max}$ is the number of binding sites under saturated conditions. **b**, $K_a$ was calculated by the quotient of $^{125}$I-1C5F5-bound/$^{125}$I-1C5F5-free as compared to the amount of $^{125}$I-1C5F5-bound. ○, untreated RMB-1 cells; $B_{max}$ 180,000 cell$^{-1}$; $K_a$ $7.4\times10^8$ l mol$^{-1}$. ●, RMB-1 cells cultured with IFN-$\gamma$ for 24 h; $B_{max}$ 335,0000 cell$^{-1}$; $K_a$ $4.2\times10^8$ l mol$^{-1}$. ▲, RMB-1 cells cultured with IFN-$\gamma$ for 48 h; $B_{max}$ 218,000 cell$^{-1}$; $K_a$ $3.6\times10^8$ l mol$^{-1}$.

**Figure 6** Effect of IFN-$\gamma$ on the immunogenicity of RMB-1 cells. BALB/c mice were s.c. immunised with $3\times10^7$ RMB-1 cells that had been cultured with IFN-$\gamma$ for 24 or 48 h. As a control mice were immunised with RMB-1 cells that had been cultured in the absence of IFN-$\gamma$. Seven days later all mice were challenged for DTH with $6\times10^6$ similar RMB-1 cells. Each column represents the arithmetic mean of the response $\pm$ 1 s.e.m. ($n=5$).

might also give rise to the immunosuppression. The retroviral protein p15E should particularly be mentioned in this respect (Bendinelli *et al.*, 1985).

When suppression can be induced by a huge amount of tumour antigens presented to the immune system, this phenomenon might play a role in clinical practice, since irradiation is frequently used in the therapy of malignant tumours, which leads to massive tumour reduction, and therefore to the appearance of large amounts of tumour-derived antigens in the bloodstream. This in turn could lead to suppression of the induction of an adequate immune response.

One approach to achieving tumour rejection is to prevent or block a possible immunosuppressive mechanism. Alternatively one might enhance the host's anti-tumour response using adjuvants, such as BCG (Hawrylko, 1980) and *Corynebacterium parvum* (Dye *et al.*, 1981). A third way would be the enhancement of tumour immunogenicity. Suda *et al.* (1986) reported increased anti-tumour immunity using haptenated tumour cells.

**Figure 7** FACScan analysis of RMB-1 cells. **a**, MHC class I expression determined using the MAb M1/42. ---- RMB-1 cells cultured with IFN-γ for 24 h, incubated with NMS as a control; —— RMB-1 cells cultured in the absence of IFN-γ, stained with the MAb M1/42; ····· RMB-1 cells cultured with IFN-γ for 24 h, stained with the MAb M1/42. **b**, MHC class II expression determined using the MAb M5/114. ---- RMB-1 cells cultured with IFN-γ for 24 h, incubated with NMS as a control; —— RMB-1 cells cultured in the absence of IFN-γ, stained with the MAb M5/114; ····· RMB-1 cells cultured with IFN-γ for 24 h, stained with the MAb M5/114.

Here we show that IFN-γ can temporarily enhance the expression of tumour-specific antigens. Interestingly, in parallel the *in vivo* anti-tumour DTH response was enhanced (Figure 6). On one hand, the enhanced DTH response could be caused by the increased expression of R-MuLV-encoded antigens. On the other hand, it is also possible that the increased MHC class I antigen expression accounts for the increased DTH response, since the immune recognition of syngeneic tumour cells may be MHC class I restricted similarly to DTH to minor histocompatibility antigens (Van der Kwast, 1980).

Various authors asked attention for the immunomodulating effects of interferons (for reviews see Krim, 1980; De Maeyer-Guignard & De Maeyer, 1985). Interferons should exert their effects via at least three mechanisms, i.e. direct cytotoxicity, increased MHC antigen expression on the tumour cells and increased host-mediated anti-tumour effects. Most studies on the effect of interferons on tumour immunogenicity focus on the increase of the expression of MHC antigens on the tumour cells (King & Jones, 1983; Green & Philips, 1986). We found increased MHC class I antigen expression on the RMB-1 cells after culture with IFN-γ, whereas class II antigen expression remained absent. The present study shows that IFN-γ in addition enhances the expression of a virally encoded or induced tumour antigen. A similar finding has recently been reported by Greiner *et al.* (1987). They demonstrated the increased expression of a tumour-associated antigen on a human colon-xenograft after treatment with IFN-α. Accumulation of immature viral budding particles at the cell surface due to inhibitory action of IFN-α/β has been described before (Friedman *et al.*, 1980). Possibly, the effectiveness of MAb therapy of tumours can be improved by simultaneous treatment with a tumour antigen-enhancing lymphokine such as interferon.

We thank Dr Th.H. van der Kwast for valuable discussions, Dr H. Bril for critically reviewing the manuscript, Mr R. van de Beemd for FACScan analysis, Mr J. Brandenburg for skilful animal care and Ms J. de Goey-van Dooren and Ms G. de Korte for excellent secretarial assistance.

### References

BENDINELLI, M., MALTEUCI, D. & FRIEDMAN, H. (1985). Retrovirus-induced acquired immunodeficiencies. *Adv. Cancer Res.*, **45**, 125.

BERENDS, D., RHIJNSBURGER, E.H., VAN GAALEN, J.L.M., VAN HOUWELINGEN, G., ZONDERVAN, P.E. & DE BOTH, N.J. (1988*a*). Syngeneic monoclonal antibodies directed against Rauscher virus induced myeloid leukemic cells: isolation and characterization. *Int. J. Cancer*, **42**, 112.

BERENDS, D., VAN GAALEN, J.L.M., RHIJNSBURGER, E.H. and 4 others (1988*b*). II. The detection of virally induced tumours by $^{131}$I- and $^{125}$I-labelled syngeneic monoclonal antibodies. *Cancer Immunol. Immunother.*, **26**, 243.

BERENDS, D., MULDER, A.H., VAN HOUWELINGEN, G. & DE BOTH, N.J. (1988*c*). III. Use of syngeneic monoclonal antibodies in the therapy of disseminated myeloid leukemic cells. *Int. J. Cancer*, **42**, 42.

BERENDS, D., VAN DER KWAST, TH.H., DE BOTH, N.J. & MULDER, P.G.H. (1989). Factors influencing antibody mediated cytotoxicity during the immunotherapy of Rauscher virus induced myeloid leukemic cells. *Cancer Immunol. Immunother.*, **28**, 123.

BHATTACHARYA, A., DORF, M.E. & SPRINGER, T.A. (1981). A shared alloantigenic determinant on Ia antigens encoded by the I-A and I-E subregions: evidence for I region gene duplication. *J. Immunol.*, **127**, 2488.

BIANCHI, A.T.J., HUSSAARTS-ODIJK, VAN DER KWAST, TH.H., BRIL, H. & BENNER, R. (1984). Suppression of anti-graft immunity by preimmunization. II. Characterization of the suppressor cells. *Transplantation*, **37**, 490.

BOOKMAN, M.A., SWERDLOW, R. & MATIS, L.A. (1987). Adoptive chemoimmunotherapy of murine leukemia with helper T lymphocyte clones. *J. Immunol.*, **139**, 3166.

CROWLE, A.J. (1975). Delayed hypersensitivity in the mouse. *Adv. Immunol.*, **20**, 197.

DE BOTH, N.J., VERMEY, M., VAN'T HULL, E. and 4 others (1978). A new erythroid cell line induced by Rauscher murine leukemia virus. *Nature*, **272**, 626.

DE BOTH, N.J., HAGEMEIJER, A., RHIJNSBURGER, E.H., VERMEY, M., VAN'T HULL, E. & SMIT, E.M.E. (1981). DMSO-induced terminal differentiation and trisomy 15 in a myeloid cell line transformed by the Rauscher murine leukemia virus. *Cell Different.*, **10**, 13.

DE BOTH, N.J., RHIJNSBURGER, E.H. & VAN EWIJK, W. (1983). A Rauscher-virus-induced T-lymphocyte cell line. Induction of differentiation under influence of dimethylsulfoxide and phorbolesters. *Int. J. Cancer*, **32**, 501.

DE BOTH, N.J., STOOF, T.J., KRANENDONK, M., STOKER, K., VONK, W.P. & MOL, J.N.M. (1985). The induction of myeloid leukemias by Rauscher murine leukemia virus. *J. Gen. Virol.*, **66**, 909.

DE MAEYER-GUIGNARD, J. & DE MAEYER, E. (1985). Immunomodulation by interferon: recent developments. In *Interferon, vol. 6*, Gresser, I. (ed) p. 69. Academic Press: New York.

DIJKMANS, R., VOLCKAERT, G., VAN DAMME, J., DE LEY, M., BILLIAU, A. & DE SOMER, P. (1985). Molecular cloning of murine interferon gamma (MuIFN-γ) cDNA and its expression in heterologous mammalian cells. *J. Interferon Res.*, **5**, 511.

DYE, S., NORTH, J.N. & MILLS, C.D. (1981). Mechanisms of anti-tumour action of *Corynebacterium parvum*. I. Potentiated tumour-specific immunity and its therapeutic limitations. *J. Exp. Med.*, **154**, 609.

FRIEDMAN, R.M., MAHESHARI, R.K., JAY, F.T. & CZARNIECKI, C. (1980). Mechanism of interferon inhibition of viruses that bud from the plasma membrane. *Ann. NY Acad. Sci.*, **350**, 533.

GALETTO, G., LAW, L.W. & ROGERS, M.J. (1985). The Rauscher-MuLV-induced leukemia, RBL-5, bears two tumour-associated transplantation antigens expressed on distinct molecules. *Int. J. Cancer*, **36**, 713.

GREEN, W.R. & PHILIPS, J.D. (1986). Differential induction of H-2K vs H-2D class I major histocompatibility complex antigen expression by murine recombinant interferon-$\gamma$. *J. Immunol.*, **137**, 814.

GREINER, J.W., GUADAGNI, F., NOGUCHI, P. and 4 others (1987). Recombinant interferon enhances monoclonal antibody-targeting of carcinoma lesions *in vivo*. *Science*, **235**, 895.

HALLIDAY, W.J. & WEBB, M. (1969). Delayed hypersensitivity to chemically induced tumours in mice and correlation with an *in vitro* test. *J. Natl Cancer Inst.*, **43**, 141.

HAWRYLKO, E. (1980). Induction of delayed-type hypersensitivity and anti-tumour immunity by systemic BCG. *Cell. Immunol.*, **50**, 136.

HOOVER, H.C., SURDYKE, M., DANGEL, R.B., PETERS, L.C. & HANNA, M.G. (1984). Delayed cutaneous hypersensitivity to autologous tumour cells in colorectal cancer patients immunized with an autologous tumour cell: Bacillus Calmette-Guérin Vaccine. *Cancer Res.*, **44**, 1671.

KING, D.P. & JONES, P.P. (1983). Induction of Ia and H-2 antigens on a macrophage cell line by immune interferon. *J. Immunol.*, **131**, 315.

KRIM, M. (1980). Towards tumour therapy with interferons, part II. Interferons: *in vivo* effects. *Blood*, **55**, 875.

KRIPKE, M.L. (1981). Immunologic mechanisms in UV radiation carcinogenesis. *Adv. Cancer Res.*, **34**, 69.

LEVY, J.P. & LECLERC, J.C. (1977). The murine sarcoma virus-induced tumor: exception or general model in tumor immunology. *Adv. Cancer Res.*, **24**, 1.

MIZEL, S.B., DELARCO, J.E., TODARO, G.J., FARRAR, W.L. & HILKIFER, M.L. (1980). *In vitro* production of immunosuppressive factors by murine sarcoma virus-transformed mouse fibroblasts. *Proc. Natl Acad. Sci. USA*, **77**, 2205.

MORTON, D.L., MILLER, G.F. & WOOD, D.A. (1969). Demonstration of tumour-specific immunity against antigens unrelated to the mammary tumour virus in spontaneous mammary adenocarcinomas. *J. Natl Cancer Inst.*, **42**, 289.

MOSMANN, I.R. & COFFMAN, R.L. (1987). Two types of helper T-cell clone. Implications for immune regulation. *Immunol. Today*, **8**, 223.

NORTH, R.J. (1985). Down-regulation of the anti-tumour immune response. *Adv. Cancer Res.*, **45**, 1.

NOWINSKI, R.L., EMERY, S. & LEDBETTER, I. (1978). Identification of an FMR cell surface antigen associated with murine leukemia virus infected cells. *J. Virol.*, **26**, 805.

OZAWA, H., IWAGUCHI, T. & KATAOKA, T. (1986). The Lyt phenotype of the T-cells responsible for *in vivo* tumour-rejection in syngeneic mice. *Cancer Immunol. Immunother.*, **23**, 73.

PAUL, R.D. & LOPEZ, D.M. (1987). Induction of 'innocent bystander' cytotoxicity in nonimmune mice by adoptive transfer of L3T4$^+$, Lyt-1$^+$2$^-$ mammary tumour immune T-cells. *Cancer Res.*, **47**, 1105.

ROGERS, M.J., GALETTO, G., HEARING, K.I., SIWARSKI, D.F. & LAW, L.W. (1984). Purification of a glycoprotein bearing a tumour transplantation antigen specific for Friend, Moloney, and Rauscher MuLV-induced tumours. *J. Immunol.*, **132**, 3210.

SCATCHARD, G. (1949). The attraction of proteins to small molecules and ions. *Ann. NY Acad. Sci.*, **51**, 660.

SPRINGER, T. (1980). Cell-surface differentiation in the mouse. Characterization of 'jumping' and 'lineage' antigens using xenogeneic rat monoclonal antibodies. In *Monoclonal Antibodies*, Kennett, R.H., McKearn, T.J. & Bechtol, K.B. (eds) p. 185, Plenum Press: New York.

STEFANINI, M., REUSER, A. & BOOTSMA, D. (1982). Isolation of Chinese hamster ovary cells with reduced unscheduled DNA synthesis after UV irradiation. *Somat. Cell Genet.*, **8**, 635.

SUDA, T., FUJIWARA, H., MIZUSHIMA, Y., SHEARER, G.M. & HAMAOKA, T. (1986). Augmentation of anti-tumour immune response by trinitrophenyl (TNP)-reactive helper T-cells: enhanced induction of tumour specific Lyt-1$^+$2$^-$ T-cell mediated delayed-type hypersensitivity from spleen cells of tumour-bearing mice by TNP helpers. *J. Natl Cancer Inst.*, **77**, 1267.

TANAKA, K., HAYASHI, H., HAMADA, C., KHOURY, G. & JAY, G. (1986). Expression of major histocompatibility complex class I antigens as a strategy for the potentiation of immune recognition of tumour cells. *Proc. Natl Acad. Sci. USA*, **83**, 8723.

VAN EWIJK, W., VAN SOEST, P.L. & VAN DEN BERGH, G.J. (1981). Fluorescence analysis and anatomic distribution of mouse T lymphocyte subsets defined by monoclonal antibodies to the antigens Thy-1, Lyt-1, Lyt-2 and T-200. *J. Immunol.*, **127**, 2594.

VAN DER KWAST, TH.H. (1980). H-2 restricted recognition of minor histocompatibility antigens in delayed-type hypersensitivity. *J. Immunogen.*, **7**, 315.

VAN DER KWAST, TH.H., BIANCHI, A.T.J., BRIL, H. & BENNER, R. (1981). Suppression of anti-graft immunity by preimmunization. I. Kinetic aspects and specificity. *Transplantation*, **31**, 79.

WARNER, N.L., MOORE, A.S. & METCALF, D. (1969). A transplantable myelomonocytic leukemia in BALB/c mice: cytology, karyotype, and muramidase content. *J. Natl Cancer Inst.*, **43**, 963.